# Solid Dosage Forms of Biopharmaceuticals in Drug Delivery Systems Using Sustainable Strategies

**DOI:** 10.3390/molecules26247653

**Published:** 2021-12-17

**Authors:** Clarinda Costa, Teresa Casimiro, Maria Luísa Corvo, Ana Aguiar-Ricardo

**Affiliations:** 1 LAQV-REQUIMTE, Department of Chemistry, NOVA School of Science and Technology, Universidade NOVA de Lisboa, 2829-516 Caparica, Portugal; cid.costa@campus.fct.unl.pt (C.C.); teresa.casimiro@fct.unl.pt (T.C.); 2 Instituto de Investigação do Medicamento (iMed.ULisboa), Faculdade de Farmácia, Universidade de Lisboa, Avenida Professor Gama Pinto, 1649-003 Lisboa, Portugal; lcorvo@ff.ulisboa.pt

**Keywords:** biopharmaceuticals, solid dosage forms, drying technologies, sustainable engineering, supercritical carbon dioxide, supercritical carbon dioxide-assisted spray-drying

## Abstract

Drug delivery systems (DDS) often comprise biopharmaceuticals in aqueous form, making them susceptible to physical and chemical degradation, and therefore requiring low temperature storage in cold supply and distribution chains. Freeze-drying, spray-drying, and spray-freeze-drying are some of the techniques used to convert biopharmaceuticals-loaded DDS from aqueous to solid dosage forms. However, the risk exists that shear and heat stress during processing may provoke DDS damage and efficacy loss. Supercritical fluids (SCF), specifically, supercritical carbon dioxide (scCO_2_), is a sustainable alternative to common techniques. Due to its moderately critical and tunable properties and thermodynamic behavior, scCO_2_ has aroused scientific and industrial interest. Therefore, this article reviews scCO_2_-based techniques used over the year in the production of solid biopharmaceutical dosage forms. Looking particularly at the use of scCO_2_ in each of its potential roles—as a solvent, co-solvent, anti-solvent, or co-solute. It ends with a comparison between the compound’s stability using supercritical CO_2_-assisted atomization/spray-drying and conventional drying.

## 1. Introduction

Biopharmaceuticals include enzymes, nucleic acids, monoclonal antibodies, and recombinant proteins that are either manufactured or isolated from biological sources for medical application [1]. Due to their greater specificity, affinity, and potency than those of small and chemically synthesized molecules, biopharmaceuticals have aroused tremendous scientific and market interest in recent years. The biopharmaceutical market represented USD 1.6 billion in 2016, increased 102% by 2008 [2] and continues to grow apace. One biopharmaceutical to recently attract attention is recombinant human deoxyribonuclease I (commercially called Dornase alfa) which is widely used in a solution as a cystic fibrosis treatment, as it reduces the viscosity and quantity of airway mucus. Recently, Earhart et al. [3] proposed the use of Dornase alfa for COVID-19 treatment. However, biopharmaceuticals can be challenging to formulate and deliver, owing to their molecular weights that can be up to 500 times that of small molecules. For example, the enzyme golimumab has a molecular weight of 147 kDa whereas quercetin weighs approximately 302 Da, which hinders permeation across biological membranes. It also presents poor biophysical stability, being susceptible to degradation or aggregation depending on the ambient moisture, temperature, or even pH, light, and shear stress [4,5]. Such challenges can be overcome by associating biopharmaceuticals to drug delivery systems [6], such as polymers [7,8,9], lipid-based nanoparticles [10,11,12], metal-organic frameworks [5,13], and dendrimers [14,15]. However, biopharmaceutical-based drug delivery systems are usually formulated in aqueous forms [16] which require storage at temperatures between 2 and 8 °C or even below −70 °C to prevent chemical and physical destabilization [17]. Nevertheless, converting these biopharmaceuticals-loaded drug delivery systems into solid dosage forms (or dry powder formulations) removes the water and minimizes oxidation and hydrolysis degradation, thereby improving stability and reducing cold distribution and supply chain costs [18]. Moreover, solid dosage conversion widens the range of potential administration routes to include intravenous, intraperitoneal, oral, nasal, and pulmonary administration. The latter is particularly attractive as the lung’s physiognomy increases bioavailability due to its large absorptive surface area. Advantages of pulmonary administration include: the ability to use smaller dosage sizes due to the local biodistribution enhancing effect, the avoidance of first-pass hepatic elimination, gut wall metabolism, and/or gastrointestinal tract destruction [19,20].

Freeze-drying is the best-known drying process for biopharmaceuticals [21,22]. Fifty percent of biopharmaceuticals approved by the U.S. Food and Drug Administration (FDA) and the European Medicines Agency (EMA) in 2016 were estimated to be freeze-dried [23]. Spray-drying and spray-freeze-drying offer greater control of particle morphology and scalability, while requiring less time. In addition, plentiful data are available for the use of these techniques in biopharmaceutical drying [16,18,24]. Exubera^®^ [25], by Pfizer, was a commercial example of insulin-based solid dosage form for diabetes treatment. This ready-to-inhale powder was mostly made up of mannitol, glycine, and sodium citrate insulin excipients that are produced by spray-drying. In 2007, Exubera^®^ was withdrawn from the market due to low sales [26]. Ibrahim et al. [27] reported using spray-drying for basic fibroblast growth factor (bFGF) as alternative asthma and chronic obstructive pulmonary disease (COPD) therapies. Such techniques, however, are costly and require high levels of energy. What is more, they might also induce heat, cold, or shear stress, resulting in biopharmaceutical degradation.

Recent decades have seen an upsurge of interest in solid dosage forms using supercritical fluids (SCF) since first reported in 1879 by Hannay and Hogarth [28]. The advantages include the ability to operate at mild conditions, their cost-effectiveness, the ability to produce microparticulate protein powders, and their scaling up feasibility [29]. Supercritical CO_2_ (scCO_2_) is the most common SCF, with a low critical temperature and moderate critical pressure at 31.1 °C and 7.38 MPa, respectively, making them suitable for processing heat-sensitive compounds [20,30]. Moreover, CO_2_ is non-flammable, non-toxic, recyclable, inert, chemically stable, and, as it is a gas at normal conditions, it can be removed by simply lowering the pressure [19]. At supercritical conditions, CO_2_ has liquid-like density and gas-like transport properties. Thus, its polarity and solvation power are superior, and it offers the mass transfer properties of a low viscosity gas in association with high associated diffusivity [31].Moreover, these properties are easily tunable near the critical point via minor temperature or pressure changes [32]. For this reason, scCO_2_ can be used with different technologies, depending on its solvating behavior. In fact, these processes can be classified into different groups depending on the scCO_2_ processing role: acting as a solvent, co-solvent, anti-solvent, or as a co-solute [33]. scCO_2_ use also respects some of the *Twelve principles of green chemistry*, outlined by Anastas in 1991 [34], rating in fifth place *Safer solvents and auxiliaries*_._ Next, we will review the latest works on the bottom-up assembly of solid dosage forms of biopharmaceuticals in drug delivery systems using scCO_2_ as a sustainable strategy.

## 2. Supercritical CO_2_-Based Drying Techniques to Produce Solid Dosage Forms of Biopharmaceuticals in Drug Delivery Systems

### 2.1. Rapid Expansion of Supercritical Solvent (RESS)

**Figure 1:**
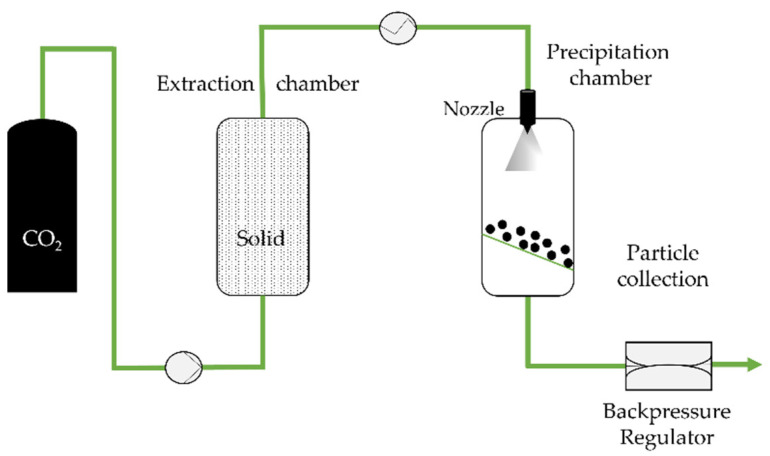
The rapid expansion of supercritical solvent (RESS) process, adapted from Aguiar-Ricardo et al. [39].

The rapid expansion of supercritical solvent (RESS) is a 1987 technique by Peterson, Matson, and Smith [35,36,37] which consists of saturating supercritical fluids with a solid substrate (and the coating agent), without any organic solvents. Upon depressurization to subcritical pressure using a heated nozzle, the substrate precipitates into a lower pressure precipitation chamber as the solution density drops (Figure 1) (usually near atmospheric pressure) [35,38].

The morphology and physicochemical properties of the resulting powders are related to the processing parameters (temperature, pressure, nozzle geometry) and the chemical structure of the material [40]. RESS has been applied mainly to the production of micronized poor water-soluble compounds. In 1993, Reverchon et al. [41] reported the micronization of salicylic acid. The first particles were in needle form, but the work was optimized, and spherical particles resulted. Years later, Yildiz and co-authors [42] reported the micronization of salicylic acid and paclitaxel, using ethanol as a co-solvent to improve salicylic acid’s solubility in CO_2_. This highlights, in fact, one of the technique’s limitations. On the one hand, scCO_2_ presents low solubility for most polymers and pharmaceuticals. On the other, the addition of a second component influences phase behavior. Unfortunately, there is little data available for the multicomponent systems involved in real processes [43].

Years later, a variation in a non-solvent RESS method (RESS-N), introduced by Mishima and co-authors, aroused considerable interest [44]. An organic solvent which does not, in its pure form, solubilize the polymer was added as a co-solvent. Nevertheless, in the presence of those organic solvents, the polymer solubility in CO_2_ increased significantly. In sum, a suspension of a drug in the ternary system composed by CO_2_, the non-toxic organic solvent (such as ethanol, isopropanol), and the dissolved polymer was sprayed through the nozzle. The polymer excipient then precipitated around the drug, producing core–shell structured microparticles [45]. Another process modification involved the rapid expansion of a supercritical solution into a liquid solvent (RESOLV) in order to obtain nanoscale particles [46,47]. In this case, however, the supercritical solution was expanded into a liquid solvent instead of a lower pressure precipitation chamber, and particles with a diameter less than 50 nm were obtained [46].

RESS is more often used with poor water-soluble compound processing, yet it (and its derivatives) has been also applied to encapsulating biopharmaceuticals in drug delivery microsystems. In fact, the processing of water-soluble compounds using RESS can be quite challenging due to CO_2_′s poor solubility in water. Table 1 displays research reporting on the encapsulation of biopharmaceuticals and the final dry powder properties.

### 2.2. Particles from Gas-Saturated Solutions (PGSS)

**Figure 2:**
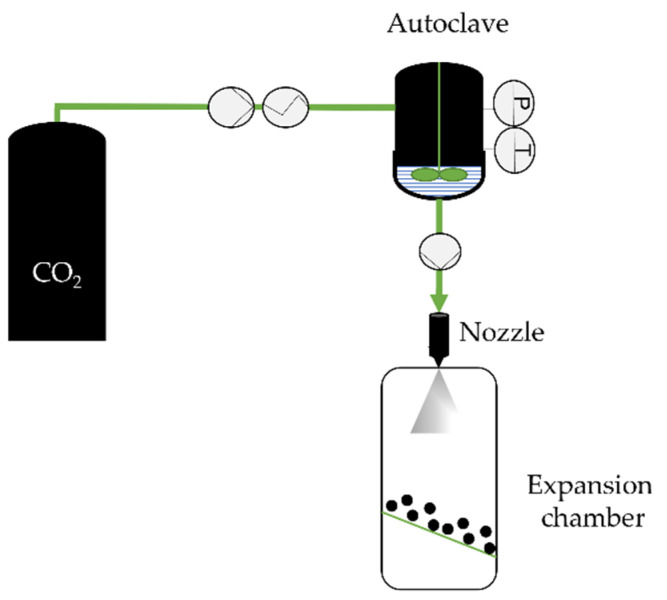
The particles from gas-saturated solutions (PGSS) technique, adapted from Kerč et al. [52].

The PGSS technique [51] was introduced in 1994 by Weidner, Knez, and Novak and uses CO_2_ as a solute. ScCO_2_ is dissolved into a melted material, consisting of drugs, polymers, biopolymers, oils, or fatty acids in a stirred high-pressure vessel until saturation is achieved [33]. The gas saturated liquid is then expanded through the nozzle to an expansion chamber at ambient pressure and lower temperature where the particles precipitate (Figure 2).

**Figure 3:**
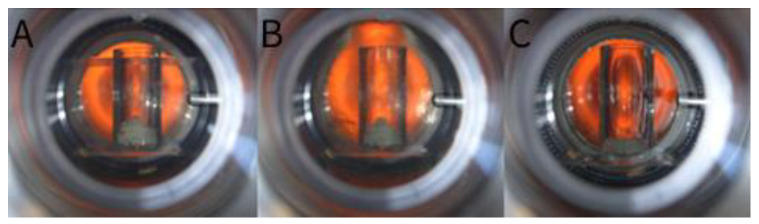
Plasticization of the P(_L_LA)-PEG_1500_-P(_L_LA) copolymers by scCO_2,_ with rise in working temperature and pressure, from Perenelli et al. [55]. (**A**) Stage conditions at 29 °C and 71 bar; (**B**) conditions at 35 °C and 85 bar; and (**C**) plasticization point at 47 °C and 140 bar. Reproduced with permission. Copyright 2014, Elsevier.

*P* and *T*–Pressure and temperature indicator and controller in the autoclave, respectively. PGSS can be more effective than RESS insofar as the compound (drug or polymer) does not need to be dissolved in scCO_2_ [52] and no organic solvents are necessary. Moreover, PGSS enables polar and water-soluble compounds to be processed. For this reason, this technique has been widely used in the pharmaceutical field with different compounds. Initially, compounds were processed without carriers. In 1999, Kerč and co-workers [52] used the technique to micronize nifedipine and felodipine (commonly used in hypertension treatment) in order to increase the dissolution rate and their bioavailability. Using temperatures between 150 °C and 185 °C, nifedipine and felodipine microparticles with an irregular shape showed a mean size between 15 and 30 µm and 42 µm, respectively. Smaller microparticles showed an increased dissolution rate. However, the effective surface area was reduced by the drug’s hydrophobicity and agglomeration. Thus, to reduce agglomeration, PEG_4000_ was added. Microparticles were produced between 50 and 70 °C since the polymer decreased the drug’s melting point. It was found that the size reduction and the drug/ PEG_4000_ interaction contributed for an enhanced dissolution rate against the pure micronized drugs. Interestingly, this study allowed the further micronization of drug delivery systems with different carriers. Hao et al. [53] produced poly(_DL_-lactic acid) (P(_DL_LA)) microparticles, affirming that solid drug particles can be mixed into the plasticized polymer. Figure 3 shows that scCO_2_ dissolves into and plasticizes the polymer at temperatures significantly below its glass transition temperature [54].

A year later, Hao et al. [56] applied PGSS to produce PEG microparticles and observed that the temperature increase and the pressure decrease favored the production of spherical particles. These works contributed to the development of the CriticalMix™—a solvent-free encapsulation process based on PGSS [57]. Rodrigues et al. [58] reported the encapsulation of theophylline—a bronchodilator—in hydrogenated palm oil, yielding microparticles sized between 2 and 3 µm. Higher working pressures (*P*) were found to lead to a more spherical size and a more limited distribution. Lower *P* of 120 or 140 bar led to agglomerated structures. Theophylline encapsulation was lower since the drug was mostly located on the carrier’s surface. More recently, Tokunaga et al. [59] reported the encapsulation of an amino acid, phenylalanine, in Eudragit L100. The work was carried out at 50 °C, between 80 and 120 bar. Microparticles sized from 200 µm to 140 µm were obtained for the respective pressures. At 100 bar, an encapsulation efficiency (*EE*) of 68.7% was achieved. In fact, the PGSS technique produced microparticles with a wide range of diameters, depending on the working parameters.

PGSS can encapsulate biopharmaceuticals at relatively low temperatures, which makes it attractive for thermolabile compound processing. Moreover, the lack of organic solvents contributes to the integrity of compounds’ activity. In 2005, Whitaker et al. [60] reported the encapsulation of ribonuclease A (RNase A), lysozyme, recombinant human insulin, and salmon calcitonin in P(_DL_LA) microparticles sized between 10 and 300 µm. Neither enzyme demonstrated an alteration. In turn, insulin activity decreased (although less so in the case of in P(_DL_LA) insulin encapsulation than for the control insulin powder). As for salmon calcitonin activity, the protein concentration was too low to measure. Salmaso et al. [61] describe an improved PGSS technique (GAMA–gas-assisted melting atomization) applied to the micronization of protein-loaded lipid particles. In this case, the protein and the melted lipid were loaded into a melting chamber that was then pressurized with scCO_2_ at 150 bar at a working temperature (*T*) of 40 °C, while undergoing stirring. The loaded lipid particles were dried with a stream of co-axially injected hot air. Vezzù et al. [62] encapsulated RNase A in lipids and polymers and observed that smaller particles were produced at lower temperatures. Table 2 reports the PGSS encapsulation of a range of biopharmaceuticals.

PGSS has also been modified to encapsulate bioactive molecules in solid lipid nanoparticles (SLN). Couto et al. [67] reported the encapsulation of Vitamin B2 into fully hydrogenated canola oil using a modified PGSS technique, where a stream of water and a stabilizer (PEG) were added to suspend SLN in water, yielding loaded SLN in aqueous bulk.

### 2.3. Carbon Dioxide-Assisted Nebulization with a Bubble Dryer (CAN-BD)

**Figure 4:**
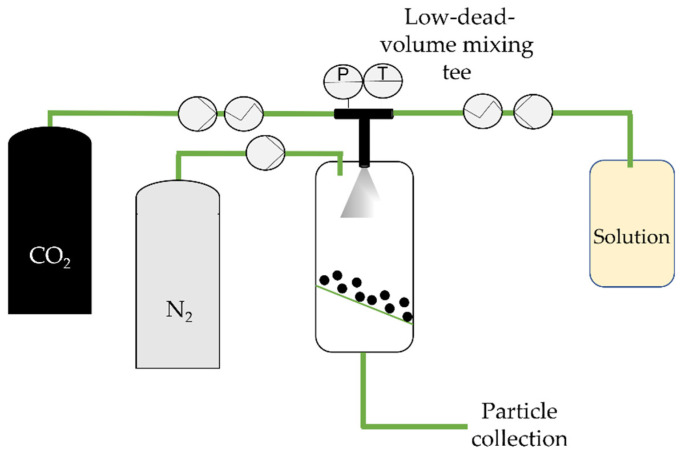
The carbon dioxide-assisted nebulization with a bubble dryer (CAN-BD) technique, adapted from Sievers et al. [69]. *P*, *T*: Pressure and temperature indicator and controller in the low-dead-volume mixing tee, respectively.

In 1997, Sievers et al. [68] proposed a PGSS improvement: the carbon dioxide-assisted nebulization with a bubble dryer (CAN-BD). This technique enables the use of any water-soluble compound, as well as the nebulization of inorganic salts or proteins [69]. Here, CO_2_ serves as a co-solute. An aqueous stream containing the drug and any excipients or stabilizers (typically 1 to 10% of the total solids dissolved [33]) is mixed with supercritical CO_2_ in a low-dead-volume mixing tee, forming a gas–liquid emulsion. This is then rapidly decompressed through a flow restrictor, forming an aerosol of droplets containing dissolved CO_2_. Upon expanding the dissolved CO_2_, the droplets rupture, and a fine aerosol, usually of diameters below 5 µm, is produced (Figure 4). It is important to point out that the expansion is enhanced since CO_2_ is one of the most soluble gases in water (1.6 mole% at 63 °C and 1500 psi). Dry particles are obtained in a furnace [70], and micronized particles are then collected on a filter.

Due to the use of the aqueous stream, CAN-BD has been widely applied to processing water-soluble compounds. In 2001, Sellers et al. [71] reported the encapsulation of proteins (lysozyme and lactate dehydrogenase) using sucrose and mannitol as excipients for pulmonary delivery. A buffer was used to neutralize any potential acidification of the solution upon the addition CO_2_ to avoid damaging the protein. The proteins were also found to maintain their activity after rehydration due to the excipients and surfactant. Years later, Sievers et al. [72] reported the encapsulation of hepatitis B surface antigen (HBsAg) and a live-attenuated measles virus by trehalose and sucrose. In addition to achieving powder stability after 43 days of storage, they were also able to provide pulmonary administration. In 2008, Cape et al. [73] reported an impressive review of the CAN-BD applied to biopharmaceutical processing. Curiously, since then, CAN-BD has been mostly used to produce vaccines, such as the Edmonston-Zagreb live-attenuated measles virus vaccine [74,75,76], and inhalable antibiotics [77]. To our knowledge, no data on biopharmaceuticals in solid dosage forms have been reported.

### 2.4. Supercritical Assisted Atomization (SAA)/Supercritical CO_2_-Assisted Spray-Drying (SASD)

**Figure 5:**
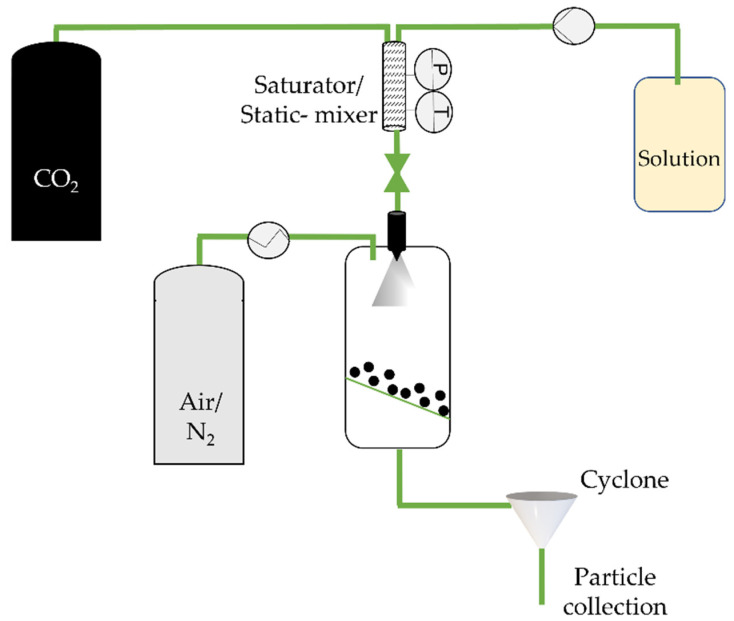
The supercritical CO_2_-assisted spray drying (SASD) apparatus at NOVA’s laboratory. *P*, *T*: Pressure and temperature indicator and controller in the static-mixer, respectively.

Supercritical assisted atomization (SAA) is based on the PGSS and CAN-BD techniques. First patented by Reverchon in 2001, it consists of the production of nano and microparticles from liquid formulations [78]. This one-step process is very similar to spray-drying but is aided by scCO_2_ and it can also be called supercritical CO_2_-assisted spray drying (SASD) [31,43]. scCO_2_ acts as a co-solute and is solubilized into a liquid solution containing the drug and/or a carrier system. The resulting near-equilibrium mixture flowing out of the saturator is atomized using a nozzle in a precipitation chamber at near atmospheric pressure, where it is dried with a hot air/nitrogen flow [43,79] which serves to avoid the Joule–Thomson effect upon decompression. Two atomization processes take place: (i) droplets at the nozzle are produced by pneumatic atomization; and (ii) CO_2_ is quickly released from inside the droplets [80]. Figure 5 illustrates a SAA/SASD process.

A series of SAA/SASD studies have been published over the years reporting microparticle production, even for poorly water-soluble compounds. In 2002, Reverchon and co-authors reported the micronization of various compounds using SAA, from salts to corticosteroids [81]. Next, several demonstrated the micronization of antibiotics for inhalation [82,83,84]. The research continued, and later polymeric microparticles (chitosan [85], poly(methyl methacrylate), and poly-_L_-lactide [86]) were produced using the technique. Reverchon, in 2007, proposed to produce a composite, in which ampicillin trihydrate and chitosan were selected as a model drug and carrier, respectively, upscaling the technique for the production of a drug delivery system [87]. Several projects have developed drug delivery systems for inhalation. Cabral et al. [88] reported the production of inhalable ibuprofen-loaded chitosan microparticles. Nano-in-microparticles were also developed for effective pulmonary drug delivery using dendrimers [89] and gold nanoparticles [90] as well as gold-coated magnetite nanocomposites [91]. As in the case of RESS, SAA/SASD has also undergone modifications, such as the introduction of a hydrodynamic cavitation mixer (HCM) [92], to improve mass transfer. Smaller diameter microparticles were reported with a narrower particle size distribution than those produced without HCM.

SAA/SASD for encapsulating and delivering biopharmaceuticals (Table 3) has been studied for a large range of compounds from proteins [30] to nucleic acids [93]. Additionally, the encapsulation of a hydrophilic dye as a molecule model (5(6)-carboxyfluorescein) has recently been encapsulated in liposomes [94] upon encapsulating trehalose using SASD. The liposomes kept their structure upon resuspension and the encapsulation efficiency was above 95%. The powders showed a mass median aerodynamic diameter of 1.75 ± 0.04 µm and a fine particle fraction of 65 ± 3%, making them fit for pulmonary administration. Storage stability assays at relative humidities of 4%, 50%, and 78% at 20 °C for 7 and 30 days then revealed that the dry powder formulations kept their liposome structural stability at relative humidities of 4% and 50% at 20 °C for 30 days.

An adaptation of SAA to produce liposomes was recently reported [103]. Supercritical assisted Liposome formation (SuperLip) is a continuous operative process, in which biopharmaceuticals can be encapsulated in liposomes, with high reproducibility [104,105]. It is important to point out that these vesicles are obtained in aqueous bulk instead of solid dosage forms.

### 2.5. Depressurization of an Expanded Liquid Organic Solution (DELOS)

**Figure 6:**
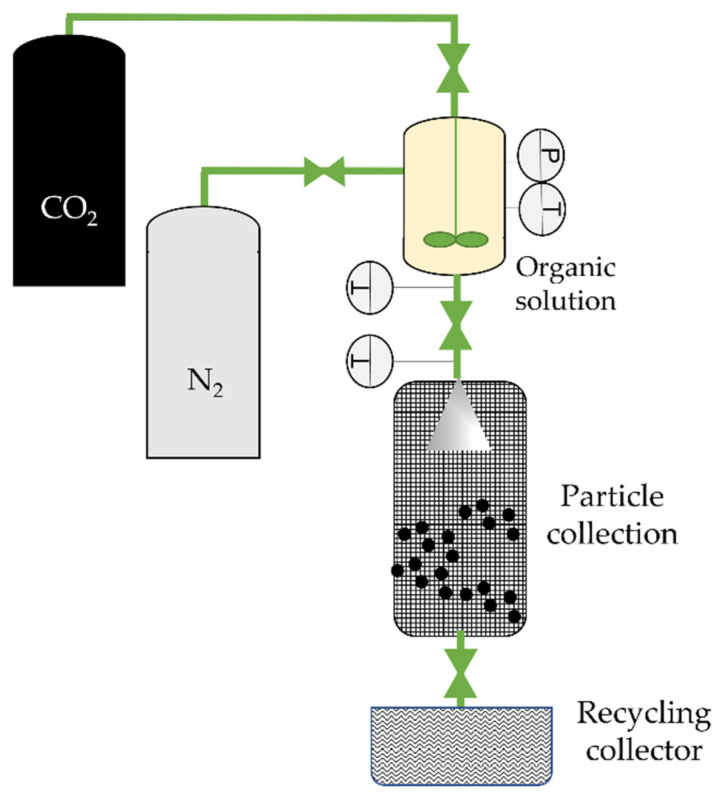
The DELOS process, adapted from Ventosa et al. [106]. *P*, *T*: Pressure and temperature indicator and controller in the high-pressure vessel and before and after the non-return valve.

Ventosa and co-workers [106] reported the depressurization of an expanded liquid organic solution (DELOS) crystallization technique twenty years ago, and it has been commonly used for producing nano- and micro-sized crystalline particles. In this process, at certain temperature and pressure conditions, a compressed gas, e.g., CO_2_, is completely miscible with the organic solvent, acting as a co-solvent, unlike in other techniques [107]. This is a three-step process. First, the solute is dissolved in the organic solvent, below the saturation limit, at working temperature and atmospheric pressure. Then, an expanded liquid mixture is obtained by adding the compressed fluid, at high working pressure, which should not exceed the critical point of the CO_2_/solvent mixture. Finally, depressurization takes place by way of using a non-returning valve upon the rapid reduction of the pressure of the expanded liquid mixture, leading to the precipitation of nano- or microparticles [33,107] (Figure 6).

Since 2001, DELOS has been applied to the micronization of polymers, surfactants, [108] and poorly water-soluble compounds such as naproxen and ibuprofen [109,110,111]. This process uses less CO_2_; the filtration takes place at atmospheric pressure, and the lack of nozzles improves scalability [112]. A technique for developing unilamellar nanovesicles based on DELOS was developed that was similar to the above-mentioned SuperLip [113]. Depressurization of an expanded liquid organic solution into aqueous solution (DELOS-susp) is mainly applied to produce nanovesicles to carry nucleic acids and enzymes [50,114,115]. However, loaded liposomes are obtained in aqueous bulk rather than in the dried form.

### 2.6. Supercritical CO_2_ as Anti-Solvent

**Figure 7:**
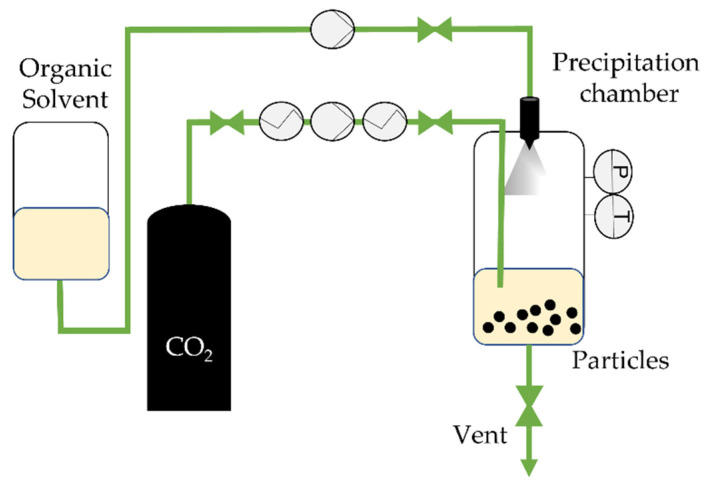
The SAS process, adapted from Aguiar-Ricardo et al. [39]. *P*, *T*: Pressure and temperature indicator and controller in the precipitation chamber, respectively.

Since scCO_2_ is a poor solvent for most pharmaceutical compounds and polymers, some approaches use it as an anti-solvent. In a supercritical anti-solvent (SAS), a solute is dissolved into an organic solvent and precipitates when scCO_2_ is added. In fact, the precipitation occurs by the diffusion of CO_2_ in the organic solvent, expanding the liquid mixture, decreasing its density, and consequently, its solvation power, until nucleation and precipitation occur [116]. The mass transfer between the CO_2_ and the organic solution decreases the surface tension, which is strong enough to control droplet shape [117]. Figure 7 illustrates this process.

The CO_2_ is pumped until a desired pressure in the heated precipitator is achieved. The organic solvent with the solute(s) is then injected by nozzle. In the case of a drug/polymer system, both precipitate under supersaturation and the drug are trapped into the polymer matrix [118]. The loaded polymer precipitates on a filter and the organic solvent/CO_2_ is recovered for further separation [119]. Impressive reviews related to pharmaceutical encapsulation using the SAS and SAS-based methods have been published [117,119]. However, the use of organic solvents to dissolve the solute may represent an obstacle to biopharmaceutical applications [118]. Even so, some research has reported successful biopharmaceutical drying with the SAS technique (Table 4). In 1993, Yeo et al. [120] reported the first production of microparticulate insulin powders using SAS, obtaining powders with diameters mostly less than 4 µm. The blood glucose level decreased with the administration of the insulin microparticles. Further results showed that SAS can compromise proteins’ secondary structure, although precipitated insulin recovered its native structure upon redissolution in aqueous media [121]. Some modifications of the SAS process have been proposed to overcome this drawback, where CO_2_ also acts as an anti-solvent. However, this review reported little focused attention on this aerosol solvent extraction system (ASES) [122] using solution-enhanced dispersion by supercritical fluids (SEDS) [123], precipitation with a compressed fluid anti-solvent (PCA) [124] and SCF-assisted extraction of emulsions (SFEE) [119,125].

## 3. Biopharmaceutical Stability during Freeze-Drying, Spray-Drying, and Supercritical CO_2_-Assisted Atomization/Spray-Drying

Biopharmaceutical stability after processing is an interesting drying issue. Despite the advantages of freeze-drying, including the freedom from the need for high temperatures and moisture control, biopharmaceuticals are submitted to different stresses, such as crystallization, dehydration changes, ionic strength changes, interfacial stress, and ice crystal formation [24]. In fact, the drying of enzymes and proteins without excipients can lead to dimers and aggregates [130]. Zhai et al. [131] reported a herpes simplex virus 2 activity recovery of 22% for a minimum number of excipients, whereas a higher recovery (80%) was found when higher percentages of excipients were used. However, recent studies have shown that the effectiveness of the excipients on biopharmaceutical stabilization depends mainly on the protein/enzyme structure [132]. For this reason, the solute concentration and solute–excipient–buffer interaction should be considered for a stable biopharmaceutical solid dosage form, using freeze-drying. Spray-drying is an attractive technique for biopharmaceuticals insofar as it is simple and easy to scale-up, plus it offers particle size control and good aerosolization. However, the biopharmaceutical is subject to thermal, atomization, interfacial, and mechanical stresses [24]. Ajmera et al. [133] reported that the combination of two amino acids—arginine and glycine—was mandatory for catalase stability during spray-drying. Without excipients, the catalase lost around 50% of its activity due to the destruction of hydrophobic interactions during drying and the breakup of intermolecular hydrogen bonds upon removing the hydration shell. With the presence of the amino acids, catalase activity increases to 60–80%. A recent study from Fernandes et al. [134] showed a SOD (*Cu*, *Zn-superoxide dismutase*) activity retention between 50–80%, with no loss of SOD conformational stability. They observed, as in the case of freeze-drying, a stabilizing effect induced by the presence of excipients (trehalose and leucine). Ji et al. [135] showed a total lysozyme bioactivity of approximately 71.5% without excipients. A notable increase in bioactivity was observed upon the addition of trehalose, Tween 20, or phosphate-buffered saline (PBS), and at a lower inlet temperature (80 °C).

Biopharmaceuticals processed with SAA/SASD are also submitted to atomization stress. However, several studies have shown that protein stability suffers only a slight change during the process. The tunable properties of scCO_2_ mean that particle engineering can speed up the process. Du et al. [99] found that an increase of *P_CO__2_* led to a loss of relative activity, due to the acidity of CO_2_, with a consequent pH change that provoked protein denaturation. The authors also observed a relative activity of 95% when the mixer was at 100 °C. It is important to call attention to the fact that no excipients were used during drying. Another study from Shen et al. [101] showed the processing of trypsin in SAA-HCM. Free trypsin, without excipients, presented a relative activity of 74.3%, whereas the relative activity of trypsin from spray-drying was 66.1%. Silva et al. [93] also reported a stable nucleic acid after SASD. It would be interesting to study SOD activity from the solid dosage SASD forms to compare with those from freeze-drying and spray-drying. All of these studies show that SAA/SASD is not merely an effective process for particle morphology control and scalability. Processing in mild conditions might also reinforce biopharmaceutical stability without compromising its conformational structure.

## 4. Conclusions and Future Perspectives

This work reviews scCO_2_-based techniques for producing solid dosage forms of biopharmaceuticals. scCO_2_ provides green and sustainable alternatives to conventional techniques, such as spray-drying for producing dry powders, as it reduces the heat and shear stress and, even when using organic solvents, their amount is lower. The encapsulation of biopharmaceuticals in solid dosage forms using RESS, PGSS, CAN-BD, SAA/SASD, DELOS, and SAS has been intensively reviewed. RESS showed good size control. However, the poor solubility of drugs and polymers in scCO_2_ hindered particle recovery leading to scale-up limitations and other disadvantages. In contrast, PGSS presents easy scalability, requires no organic solvents, and can take place at low temperatures. However, since it requires the solubilization of scCO_2_ in the melted compound, the technique is limited to pharmaceutical compounds in which CO_2_ is highly soluble. Moreover, the fact that the solute must be melted represents a potential issue for heat-sensitive materials. CAN-BD is an interesting method as a wide range of compounds can be processed, although high temperatures are required. SAA/SASD presents good particle size control, high drying and atomization efficiency, and easy tunability to produce microparticles. Yet, small amounts of organic solvents might be required to improve the drying process. Using the DELOS technique, low pressures can be applied for optimum precipitation. However, the process is not applied to biopharmaceuticals (probably since biopharmaceutical crystallization might occur). Finally, SAS was found to be attractive for processing insoluble compounds in CO_2_ (as is the case for most drugs). However, the use of organic solvents to dissolve the solute may represent an obstacle to biopharmaceutical applications. Unfortunately, throughout the literature, there is less data about producing solid dosage forms of biopharmaceuticals than that looking into the same system for poorly water-soluble and low molecular weight molecules, excepting SAA/SASD. As a matter of fact, even scCO_2_ techniques present impressive and potential results; biopharmaceuticals are very sensitive to their environment and their processing may prove more challenging. For this reason, future research into the development of nano-in-microparticles solid dosage forms is predicted to increase. Biopharmaceuticals are first encapsulated in a nanosystem that is then encapsulated in microparticles. We believe that this scCO_2_-based technique will potentially produce loaded solid dosage forms, without compromising the biopharmaceutical structure or stability.

## Figures and Tables

**Table 1:** Solid dosage form biopharmaceuticals in RESS drug delivery systems.

Year	Active Compound	Co-Solvent	Solid Dosage Form	Observations	Ref.
2000	Lysozyme * Lipase	Ethanol	PEG ^1^ PMMA ^2^ P(_DL_LA) ^3^ P(St) ^4^ PLGA ^5^ PEG-PPG ^6^	Lower diameters for ethanol as a co-solvent The size is more influenced by the polymer feed composition	[44]
2002	BSA ^7^	N/A	Dynasan^®^114 Gelucire^®^50-02	Similar RESS method Dynasan^®^114-based microparticle diameter < 50 µm 13% < BSA content < 62% Gelucire^®^50-02-based microparticles with a mean size of 543 µm 36% < BSA content < 67%	[48]
2012	Co-enzyme Q_10_	Ethanol Acetone Dichloromethane	PEG ^1^ P(_DL_LA) ^3^	Microparticle diameter between 2–8 µm PEG coating results in higher microparticles Higher coQ_10_ release for higher microparticles	[49]
2013	Insulin	Ethanol	Tripalmitin	RESS is combined with the supercritical assisted drying (SAD) 3.5 µm < PSD ^8^ < 11 µm Insulin content of 33.1%	[50]

^1^ Poly (ethylene glycol); ^2^ Poly (methyl methacrylate); ^3^ Poly (_DL_-lactic acid); ^4^ Poly (styrene); ^5^ Poly (_DL_-lactide-co-glycolide); ^6^ Poly (propylene glycol); ^7^ Bovine serum albumin; ^8^ Particle size distribution; * RESS-N.

**Table 2:** Solid dosage forms of biopharmaceuticals in PGSS drug delivery systems.

Year	Active Compound	Co-Solvent	Solid Dosage Form	Observations	Ref.
2005	RNase A Lysozyme Insulin Salmon calcitonin	N/A	P(_DL_LA)	10 µm < PS ^1^ < 300 µm Insulin and salmon calcitonin microparticles with rough morphologies Poor particle size control RNase A and lysozyme retained their enzymatic activity Stored insulin microparticles decrease in activity at 25 °C for 1 week and 1 month Due to the low dosage of calcitonin, the salmon calcitonin was mixed with polymer powder and freeze-dried	[60]
2009	Insulin Recombinant human growth hormone (rh-GH)	1 mL of DMSO ^2^ containing the protein	Tristearin/Phosphatidylcholine/PEG_5000_	Spherical particles with a mean diameter of 197 nm Insulin recovery of 57 ± 8% rh-GH recovery 48 ± 5% Glucose reduction of 50% with the lower dose and 70% at the higher dose, in 1–2 h.	[61]
2009	Insulin	1 mL of DMSO containing the protein	Tristearin/Phosphatidylcholine/PEG/Tween80 Tristearin/Phosphatidylcholine/dioctyl sulfosuccinate	The authors defined the process as GAMA Binodal size distribution two main particle size populations. The main fraction had a diameter range of 200–400 nm, and a minor fraction had a diameter range of 80–120 nm.	[63]
2010	RNase A	1 mL of DMSO containing the enzyme	Tristearin/Phosphatidylcholine/PEG_5000_	The higher the *T*, the higher the product yield The higher the *T*, the higher the particle size *EE* up to 80% The enzyme retained its a residual activity of about 83%	[62]
2010	Human growth hormone (hGH)	N/A	P(_DL_LA) PLGA Excipients ^3^	Rounded particles with few pores Apparent size around 93 μm 56.1 μm < D_50_^4^ < 104.5 μm 97.1% < *EE* < 100%	[57]
2011	Co-enzyme Q_10_	N/A	PEG_6000_	Particle size of 190 nm 220 nm < Dv_50_^5^ < 2.36 µm Enzyme recovery yield of 89.8% ^6^	[64]
2011	Human growth hormone (hGH)	N/A	PLGA P(_DL_LA) Poloxamer 407	*EE* of 98.3 ± 4.6% The structural integrity of hGH is unaffected by scCO	[65]
2013	Progesterone (PGN)	N/A	PEG_400_/PEG_4000_ (50:50) D-α-tocopheryl PEG_1000_ succinate (TPGS) Gelucire 44/14	At *T* of approximately 56 °C, process yields of 95.7% for PNG-loaded TPGS86.3% for PNG-loaded PEG93.3% for PNG-loaded Gelucire 44/14 PGN showed high dissolution rates for all the formulations	[66]
2014	Bovine serum albumin (BSA)	N/A	P(_L_LA) -PEG_1500_- P(_L_LA)	19. 07 µm < PS_50_ < 78.63 µm 29.19% < Process yield < 41.74% 96.85% < *EE* < 101.75%	[55]

^1^ Particle size; ^2^ Dimethyl sulfoxide; ^3^ Poloxamer 188, poloxamer 407, or Solutol HS15; ^4^ Diameters at 50% cumulative volume; ^5^ Volume median diameter; ^6^ After PEG removal.

**Table 3:** Solid dosage forms of biopharmaceuticals in drug delivery systems produced by SAA/SASD methods.

Year	Active Compound	Nanocarrier	Co-Solvent	Solid Dosage Form	Observations	Ref.
2009	Lysozyme	N/A	Ethanol	N/A	Spherical microparticles 1.0 µm < PSD < 4.0 µm Lysozyme remained stable with biological activity from 95% to 100%.	[80]
2009	Lysozyme Trypsin	N/A	N/A	N/A	80% of trypsin and 65% of lysozyme particles have a diameter smaller than 5 µm	[95]
2010	Gentamicin sulfate *	N/A	N/A	BSA	Mean diameter of 2 µm 1.70 µm < D_50_ > 2.24 µm *EE* > 95.6%	[96]
2011	BSA	N/A	N/A	N/A	Well-defined, hollow, and spherical BSA microparticles 0.3 µm < PSD < 5.0 µm	[97]
2011	BSA	N/A	N/A	N/A	The solubility of BSA is dependent on processing temperature	[98]
2011	Lysozyme	N/A	Ethanol	N/A	SAA-HCM ^1^ 0.2 µm < PS < 5.0 µm Lysozyme kept 85% of its activity	[99]
2013	Insulin	N/A	N/A	N/A	SAA-HCM 0.5 µm < PS < 5.0 µm	[100]
2015	Trypsin	N/A	N/A	Chitosan	SAA-HCM 0.2 µm < PS < 4.0 µm *LE* ^2^ up to 91.8% Trypsin retained > 70% of its enzymatic activity	[101]
2017	BSA	N/A	Acetonitrile	PLGA	1.7 µm < MMAD ^3^ < 3.5 µm FPF ^4^ of 43% BSA showed both chemical and structural stability	[30]
2018	Parathyroid hormone	N/A	N/A	Chitosan oligosaccharide	SAA-HCM 1.0 µm < MMAD < 5.0 µm FPF of 63.51% *LE* up to 92.8%	[102]
2020	SiRNA ^5^	Mesoporous silica nanoparticles Poly-L-arginine Hyaluronic acid	Ethanol	Chitosan (CHT)	3.0 µm < D_v,50_ < 4.0 µm FPF of 44.4% *EE*_siRNA_ of 11.4% onto LBL nanosystems Entrapment efficiency of the LbL nanoparticles of 28.7% in CHT powder 90% of gene silencing from CHT-LbL siRNA	[93]

^1^ Supercritical fluid assisted atomization introduced by a hydrodynamic cavitation mixer; ^2^ Loading efficiency; ^3^ Fine particle fraction; ^4^ Mass median aerodynamic diameter; ^5^ Small interfering RNA. * This work was selected since BSA was used as a microcarrier.

**Table 4:** Solid dosage forms of biopharmaceuticals in SAS drug delivery systems.

Year	Active Compound	Co-Solvent	Solid Dosage Form	Observations	Ref.
1993	Insulin	DMSO DMFA ^1^	N/A	90% of the particles with a diameter smaller than 4 µm 10% of the particles with a diameter smaller than 1 µm Blood glucose level decreases over the time	[120]
1999	Lysozyme	DCM ^2^	(a) P(_L_LA) ^3^ (b) PLGA	PCA (a) 250 µm < Diameter < 500 µm (b) 5 µm < Diameter < 60 µm CO_2_ at high velocity through an annular region in a coaxial nozzle results in spherical and uniform particles	[124]
2001	Insulin	(a) DCM (b) DCM- DMSO (50:50,%*v*/*v*)	P(_L_LA) ^3^	(a) 1 µm < Diameter < 3 µm (b) 0.5 µm < Diameter < 2 µm	[118]
2009	Lysozyme	Water/EtOH	N/A	SEDS Fiber formation at higher pressures 0.1 µm < PSD < 0.4 µm Lysozyme activity was recovered from all spherical particles	[126]
2009	BSA	DCM	P(_L_LA) ^3^	PS < 2.5 µm The secondary BSA structure is not affected BSA content of 17.11%	[127]
2009	Lysozyme	DMSO	N/A	PCA: PS < 100 nm GAS: 233 nm < PS < 302 nm Formation of the lysozyme particles involves spinodal decomposition	[128]
2012	Insulin	DMSO/Acetone	HPMCP ^4^	138 nm < PS < 342 nm *EE* up to 100% 10. 76% < Insulin loading < 16.04%	[129]

^1^ N-N-Dimethylformamide; ^2^ Dichloromethane; ^3^ Poly-(_L_-lactide); ^4^ Hydroxypropyl methyl cellulose phthalate.
